# Impact of demographic, clinical, and treatment compliance characteristics on quality of life of Venezuelan patients with systemic lupus erythematosus

**DOI:** 10.1186/s41927-021-00232-0

**Published:** 2022-01-04

**Authors:** Fhabián S. Carrión-Nessi, María V. Marcano-Rojas, Sinibaldo R. Romero Arocha, Daniela L. Mendoza Millán, David A. Forero-Peña, Allen W. Antuarez-Magallanes, Soham Al Snih, Martín A. Rodríguez, Yurilís J. Fuentes-Silva

**Affiliations:** 1 “Dr. Francisco Battistini Casalta” Health Sciences School, University of Oriente – Bolivar Nucleus, Ciudad Bolivar, Venezuela; 2 Biomedical Research and Therapeutic Vaccines Institute, Ciudad Bolivar, Venezuela; 3 Internal Medicine Department, “Tcnel. Dr. César Bello D’Escrivan” Medical Assistance Nucleus, Ciudad Bolivar, Venezuela; 4 Medical Scientist Training Program (MD/PhD), University of Minnesota Medical School, Minneapolis, MN USA; 5 “Luis Razetti” School of Medicine, Central University of Venezuela, Caracas, Venezuela; 6 Division of Rehabilitation Sciences, University of Texas Medical Branch, Galveston, TX USA; 7 Sealy Center of Aging, University of Texas Medical Branch, Galveston, TX USA; 8 Centro Clínico Universitario de Oriente, Ciudad Bolivar, Venezuela

**Keywords:** Systemic lupus erythematosus, Health-related quality of life, Disease activity, Treatment compliance, Venezuela

## Abstract

**Background:**

We have here assessed the impact of demographic, clinical, and treatment compliance characteristics on health-related quality of life (HRQoL) of Venezuelan patients with systemic lupus erythematosus (SLE). We have used a disease-specific questionnaire, the Lupus Quality of Life (LupusQoL), validated in our patient population, to measure HRQoL.

**Methods:**

A cross-sectional study was conducted among 100 patients with SLE from outpatient clinics. Patients completed a form with demographic, clinical, and treatment compliance data, and the LupusQoL questionnaire. HRQoL was classified as *better* or *worse* according to previously established cut-off points for this patient population. Spearman’s *r* test was used to determine the correlations between age, years of education, disease duration, SLEDAI, and SLICC-DI with the eight domains of the LupusQoL. Mann–Whitney *U* test was used to compare the HRQoL between the two groups of patients according to treatment compliance. Binomial logistic regression using the backward stepwise selection method was performed to identify the risk factors associated with each of the eight domains of the LupusQoL among patients with inactive (SLEDAI < 4) and active (SLEDAI ≥ 4) SLE.

**Results:**

HRQoL of our patients was classified as *better* in all domains of the LupusQoL. Age correlated negatively with all domains of the LupusQoL, except with “burden to others”, and disease activity correlated negatively with all domains of the LupusQoL, except with “intimate relationships” and “burden to others” (*p* < 0.05). Patients who fully complied with indicated treatment had higher scores in “physical health” domain compared to patients who did not comply with at least one of the prescribed medications (*p* < 0.05). In patients with active SLE, a risk factor associated with *worse* “planning” and “intimate relationships” was advanced age, while having had SLE flare-ups in the previous six months was a risk factor associated with *worse* “physical health” (*p* < 0.05).

**Conclusion:**

Age and disease activity were negatively correlated with almost all domains of the LupusQoL, and treatment compliance was associated with higher score in the “physical health” domain. Disease control and treatment compliance should be the main goals for a *better* HRQoL in our patients with SLE.

## Background

Systemic lupus erythematosus (SLE) is a chronic autoimmune disease of unknown aetiology with variable multisystem clinical manifestations. It is highly prevalent among young women of childbearing age from African-American, Hispanic, Asian, and Caribbean ancestry [1–4]. Most patients with SLE experience unpredictable disease flare-ups that, in addition to potential drug adverse effects, can significantly worsen their health-related quality of life (HRQoL) [5, 6]. In previous studies, remission and low disease activity status were associated with better HRQoL in these patients [7–11]. Thus, HRQoL is a useful parameter to follow the management and course of the disease [12].

Patients with SLE report worse HRQoL compared to healthy individuals and similarly —or sometimes worse— to patients with other chronic diseases such as hypertension, diabetes, or myocardial infarction [13–16]. Some demographics, such as advanced age, and clinical characteristics, including high disease duration, active disease, and accrued organ damage, have been associated with impaired HRQoL in these patients [17–19]. Disease misconceptions and mood disorders caused by worse HRQoL have also been reported to worsen prognosis and treatment compliance [20]. Thus, a full assessment of the health status of patients with SLE should include an evaluation of HRQoL [21].

In the past, HRQoL of patients with SLE had been measured through generic questionnaires not designed to assess disease-specific characteristics [22]. However, in recent decades, the use of disease-specific questionnaires to measure HRQoL has gained great interest due to their superior sensitivity to change and to the effect of treatment [23]. These new research tools have identified variations in patient functioning, increased prognosis accuracy and established reference guidelines for future cases [24, 25]. The Lupus Quality of Life (LupusQoL) is a valid, reliable, patient-derived, disease-specific HRQoL measure that includes the most relevant domains for patients with SLE; it contains eight domains: physical health, pain, planning, intimate relationships, burden to others, emotional health, body image, and fatigue [26]. The LupusQoL has good internal reliability (Cronbach’s α: 0.88–0.95), good test–retest reliability (*r* = 0.72–0.93), and good concurrent validity comparable with the domains of the Medical Outcome Survey Short Form 36 (*r* = 0.71–0.79); it also has acceptable ceiling effects and minimal floor effects [26].

Generic questionnaires have frequently been used in national [19, 27] and international [28–33] studies to measure HRQoL of patients with SLE. However, reports on this subject are scarce in Latin America. The Peruvian Almenara Lupus Cohort assessed 277 patients using the LupusQoL and found that higher socioeconomic status, shorter disease duration, and use of antimalarials were positively associated with HRQoL [34]. HRQoL has yet to be studied using a disease-specific questionnaire in Venezuelan patients with SLE. The objectives of this study were to measure HRQoL of Venezuelan patients with SLE using the LupusQoL and to assess the impact of demographic, clinical, and treatment compliance characteristics on HRQoL of these patients. We hypothesised that patients with advanced age, active disease, or who do not comply with treatment will be more likely to worse HRQoL in all domains of the LupusQoL than those who are young, without disease activity, or who comply with treatment.

## Methods

### Patients and study design

**Fig. 1:**
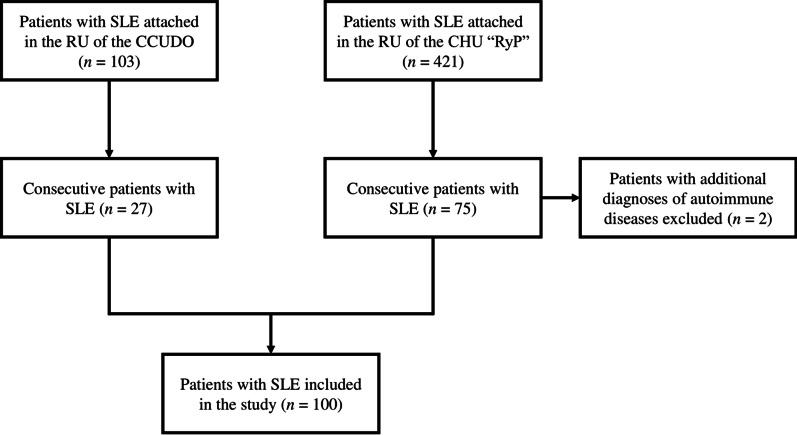
Flowchart of the patients’ selection. *SLE* systemic lupus erythematosus, *RU* Rheumatology Unit, *CCUDO* Centro Clínico Universitario de Oriente, *CHU “RyP”* Complejo Hospitalario Universitario “Ruiz y Páez”

A cross-sectional study was conducted among consecutive patients with SLE from the Rheumatology Unit of the Complejo Hospitalario Universitario “Ruiz y Páez” and the Centro Clínico Universitario de Oriente in Ciudad Bolivar, Venezuela, in the period between September and December 2019 (Fig. 1). Patients with at least four of the 1982–1997 American College of Rheumatology revised criteria [35, 36] were included. Patients with additional diagnoses of autoimmune diseases other than SLE (including secondary Sjogren’s syndrome), except patients with secondary antiphospholipid syndrome, were excluded.

### Data collection

Demographics (age, sex, years of education, current academic and employment status), clinical (disease duration, disease activity, and accrued organ damage), and treatment compliance (antimalarials, corticosteroids, immunosuppressants, and/or biological therapy) data, were collected. Disease activity and accrued organ damage were measured by the Systemic Lupus Erythematosus Disease Activity Index (SLEDAI) [37] and the Systemic Lupus International Collaborating Clinics Damage Index (SLICC-DI) [38], respectively. The SLEDAI assessed disease activity in the last 10 days and includes 24 items that inform on specific clinical and immunological manifestations, with a maximum score of 105 [37]. Patients were classified into two groups according to their SLEDAI: inactive SLE (SLEDAI < 4) and active SLE (SLEDAI ≥ 4). The SLICC-DI assesses irreversible disease damage occurring since onset of lupus, ascertained by clinical assessment and present for at least six months unless otherwise stated, and includes 42 items that measure the impact of 12 domains, with a maximum score of 46 points [38].

HRQoL was measured with the LupusQoL [26] using a version translated into Spanish and validated for the Venezuelan population with SLE [39]. The LupusQoL cut-off points were generated from a latent class analysis that allowed classifying one or more unobserved (latent) classes with respect to a variable. By applying an analysis of the Bayesian Information Criterion values, two classes were found to be optimal for the questionnaire. Then, using Receptor Operational Characteristics curves, the cut-off points for each domain of the LupusQoL were identified. These cut-off points allowed to classify patients’ HRQoL into *better* or *worse* [39]. This questionnaire contains eight domains and a total of 34 items that were answered using a five-point Likert scale. The scale gives a score by domains that ranges from 0 to 100, which can be obtained through the following formula: the responses per domain are added and divided by the total number of items in that domain, the resulting value is divided by four and then multiplied by 100 [26]. Our cut-off points were 56.25 for “physical health”, 58.33 for “pain”, 66,66 for “planning”, 65,2 for “intimate relationship”, 58.33 for “burden to others”, 54.16 for “emotional health”, 70 for “body image”, and 56.35 for “fatigue” [39].

### Patient interviews

Patients were recruited during their routine rheumatology consultation. Those who agreed to participate in the study received an informed consent form, a demographic, clinical, and treatment compliance data form, and a copy of the LupusQoL to be completed at the clinical centre or taken home to be completed and delivered back within a week. Five patients who could not read or write received help to fill out their forms by the authors at the clinical centre or from their relatives if they took it home. The SLEDAI and SLICC-DI were obtained the same day the questionnaires were completed.

### Statistical analysis

Patient data were summarised by the following descriptive statistics: mean, standard deviation (SD), median, interquartile range [IQR], and/or frequency. The distribution of variables was assessed by the Kolmogorov–Smirnov test. Mann–Whitney *U* and Spearman’s *r* tests was used for variables with a non-normal distribution, and Student’s *t*-test for those with normal distribution. Pearson’s chi-squared and Fisher’s exact test were used for categorical variables. Spearman’s *r* test was used to determine the correlations between age, years of education, disease duration, SLEDAI, and SLICC-DI with the eight domains of the LupusQoL. *P *values < 0.05 were considered significant. *R* value > 0.8 was considered very strong; 0.6 to 0.79, strong; 0.4 to 0.59, moderate; 0.2 to 0.39, weak; and < 0.2, absent. Mann–Whitney *U* test was used to compare the HRQoL between the two groups of patients according to treatment compliance: group 1 included those who fully complied with indicated treatment and group 2 included those who did not comply with indicated treatment in at least one of the prescribed medications. Binomial logistic regression using the backward stepwise selection method was to identify the risk factors associated with each of the eight domains of the LupusQoL among patients with inactive (SLEDAI < 4) and active (SLEDAI ≥ 4) SLE. The best valid model that classified the highest percentage of patients analysing its goodness of fit, R^2^ Nagelkerke, and Hosmer–Lemeshow test was taken into account. Statistical analysis was performed using Statistical Package for the Social Sciences version 26 (International Business Machines Corporation, Armonk, NY, United States). Spidergram was generated using Microsoft® Excel® version 2019 (Microsoft, Redmond, WA, United States).

## Results

### Characteristics of patients with SLE

One hundred patients met the inclusion criteria. The mean age, median years of education, and median disease duration of patients with SLE were 43 (SD—standard deviation—14), 11 [IQR—interquartile range—5], and 8 [IQR 12] years, respectively; most were women (93%), unemployed (62%), and did not comply with treatment in at least one of the prescribed medications (63%) (Table 1). Sixty-four patients had inactive SLE. Patients with active SLE (36%) had higher accrued organ damage score (*p* < 0.001) and higher prednisone indication (79.7% vs. 94.4%, *p* = 0.047).

**Table 1:** Demographic, clinical, and treatment compliance characteristics of patients with inactive and active SLE

	All (*N* = 100)	Inactive^a^ (*N* = 64)	Active^b^ (*N* = 36)	*p* value
*Demographic characteristics*				
Age, mean (SD), years	43 (14)	42 (13)	45 (14)	0.175^*^
Sex, women/men (%)	93/7 (93/7)	61/3 (95.3/4.7)	32/4 (88.9/11.1)	0.424^†^
Education, median [IQR], years	11 [5]	11 [5]	11 [9]	0.754^§^
Current academic status, studying/not studying (%)	12/88 (12/88)	8/56 (12.5/87.5)	4/32 (11.1/88.9)	1^†^
Current employment status, employed/unemployed (%)	38/62 (38/62)	27/37 (42.2/57.8)	11/25 (30.6/69.4)	0.349^†^
*Clinical characteristics*				
Disease duration, median [IQR], years	8 [12]	7 [12]	10 [15]	0.835^§^
Disease activity (SLEDAI), median [IQR], points	2 [5]	0 [1]	6 [6]	< 0.001^§^
Accrued organ damage (SLICC-DI), median [IQR], points	0 [1]	0 [0]	1 [2]	< 0.001^§^
*Treatment compliance*				
Indicated-treatment compliance, yes/no (%)	37/63 (37/63)	27/37 (42.2/57.8)	26/10 (72.2/27.8)	0.152†
Antimalarial indicated, yes/no (%)	90/10 (90/10)	59/5 (92.2/7.8)	31/5 (86.1/13.9)	0.532^†^
Compliance, yes/no (%)	56/34 (62.2/37.8)	41/18 (69.5/30.5)	15/16 (48.4/51.6)	0.094^||^
Corticosteroid indicated, yes/no (%)	85/15 (85/15)	51/13 (79.7/20.3)	34/2 (94.4/5.6)	0.047^||^
Compliance, yes/no (%)	60/25 (70.6/29.4)	39/12 (76.5/23.5)	21/13 (61.8/38.2)	0.047^‡^
Immunosuppressant indicated, yes/no (%)	45/55 (45/55)	32/32 (50/50)	13/23 (36.1/63.9)	0.180^||^
Compliance, yes/no (%)	18/27 (40/60)	14/18 (43.7/56.3)	4/9 (30.8/69.2)	0.305^||^
Biological therapy indicated, yes/no (%)	5/95 (5/95)	3/61 (4.7/95.3)	2/34 (5.6/94.4)	1^†^
Compliance, yes/no (%)	0/5 (0/100)	0/3 (0/100)	0/2 (0/100)	–
SLE flare-ups in the previous six months, yes/no (%)	33/67 (33/67)	17/47 (26.6/73.4)	16/20 (44.4/55.6)	0.068^||^
Merited hospitalisation, yes/no (%)	1/32 (3/97)	0/17 (0/100)	1/15 (6.2/93.8)	0.092^†^

*SD* standard deviation, *IQR* interquartile range, *SLEDAI* Systemic Lupus Erythematosus Disease Activity Index, *SLICC-DI* Systemic Lupus International Collaborating Clinics Damage Index, *SLE* systemic lupus erythematosus

^a^Inactive SLE was defined by a SLEDAI < 4

^b^Active SLE was defined by a SLEDAI ≥ 4

*Independent-samples Student’s *t*-test

^†^Yates’ chi-square test

^‡^Fisher’s exact test

^§^Median test

^||^Pearson’s chi-square test

### HRQoL and clinical characteristics of patients with SLE

HRQoL of patients with SLE was classified as *better* in all domains of the LupusQoL according to previously established cut-off points for the Venezuelan population with SLE [39]. Table 2 shows the Spearman’s *r* correlation coefficient between demographic, clinical, and treatment compliance characteristics with the eight domains of the LupusQoL, as well as median [IQR] of the LupusQoL scores for the two treatment compliance groups. There was moderate correlation between age and “intimate relationships” domain (− 0.43), and weak correlations between age and remaining domains, except “burden to others” (− 0.17). Likewise, there was weak correlation between years of education and “physical health” and “intimate relationships” domains (0.21 and 0.25, respectively), and between disease duration and “physical health” (− 0.34), “pain” (− 0.33), and “fatigue” (− 0.20) domains. Accrued organ damage was weakly correlated with all domains of the LupusQoL, except with “intimate relationships” and “burden to others” (− 0.12 and − 0.13, respectively) (Table 2). Patients who fully complied with treatment (*N* = 37) had higher scores in “physical health” domain compared to patients who did not comply with treatment in at least one of the prescribed medications (*N* = 63) (*p* < 0.05) (Table 2).

**Table 2:** Correlations between the demographic, clinical, and treatment compliance characteristics with the eight domains of the LupusQoL in patients with SLE

LupusQoL domains	LupusQoL score, median [IQR]	Age	Years of education	Disease duration	SLEDAI	SLICC-DI	Treatment compliance	
							No, median [IQR] (*N* = 63)	Yes, median [IQR] (*N* = 37)
Physical health	73.4 [38.3]	− 0.38^‡^	0.21*	− 0.34^‡^	− 0.32^†^	− 0.32^†^	68.8 [39.1]	84.4 [31.2]^||^
Pain	66.7 [41.6]	− 0.32^†^	0.13	− 0.33^‡^	− 0.31^†^	− 0.27^†^	58.3 [45.8]	66.7 [41.6]
Planning	83.3 [43.8]	− 0.22*	0.15	− 0.13	− 0.35^‡^	− 0.24*	83.3 [45.8]	91.7 [41.7]
Intimate relationships	75 [50]	− 0.43§	0.25*	− 0.19	− 0.15	− 0.12	75 [37.5]	75 [37.5]
Burden to others	66.7 [50]	− 0.17	0.04	− 0.01	− 0.13	− 0.13	66.7 [50]	66.7 [41.6]
Emotional health	75 [38.5]	− 0.23*	− 0.01	− 0.18	− 0.37^‡^	− 0.29^†^	70.8 [35.4]	83.3 [25]
Body image	85 [31.3]	− 0.24*	0.01	− 0.09	− 0.34^‡^	− 0.24*	80 [37.5]	90 [30]
Fatigue	62.5 [39.1]	− 0.3^†^	0.07	− 0.20*	− 0.27^†^	− 0.26^†^	56.3 [40.6]	75 [37.5]

*p < 0.05; ^†^*p* < 0.01; ^‡^*p* < 0.001; ^§^*p* < 0.0001 (*p* values by Spearman’s *r* test); ^||^*p* < 0.05 (*p* values by Mann–Whitney *U* test)

*LupusQoL* Lupus Quality of Life, *IQR* interquartile range, *SLEDAI* Systemic Lupus Erythematosus Disease Activity Index, *SLICC-DI* Systemic Lupus International Collaborating Clinics Damage Index

### Correlation between HRQoL and disease activity

**Fig. 2:**
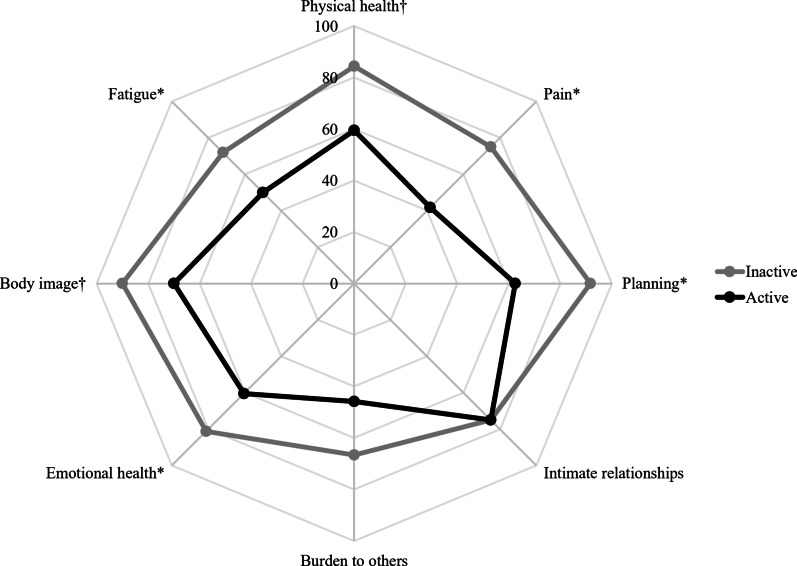
Spidergram of the eight domains of the LupusQoL in Venezuelan patients with active and inactive SLE. Data are graphed as medians. *p < 0.05; ^†^*p* < 0.01 (*p* values by median test)

Disease activity correlated negatively with all domains of the LupusQoL, except with “intimate relationships” and “burden to others” (Fig. 2; Table 2). Accordingly, patients with active SLE had significantly lower scores than patients with inactive SLE in “physical health” (59.4 vs. 84.4, *p* < 0.01), “pain” (41.7 vs. 75, *p* < 0.05), “planning” (62.5 vs. 91.7, *p* < 0.05), “emotional health” (60.4 vs. 81.3, *p* < 0.05), “body image” (70 vs. 90, *p* < 0.01), and “fatigue” (50 vs. 71.9, *p* < 0.05) domains (Fig. 2).

### Risk factors associated with HRQoL among patients with inactive and active SLE

According to the best valid model that classified the highest percentage of patients with inactive SLE in each domain, advanced age was a risk factor associated with *worse* “physical health”, “intimate relationships”, “emotional health”, and “fatigue”, while higher disease duration was a risk factor associated with *worse* “pain”. More years of education was a risk factor associated with *better* “intimate relationships”, and being employed was a risk factor associated with *better* “fatigue”. According to the best valid model that classified the highest percentage of patients with active SLE in each domain, advanced age was a risk factor associated with *worse* “planning” and “intimate relationships”, while having had SLE flare-ups in the previous six months was a risk factor associated with *worse* “physical health” (Table 3).

**Table 3:** Risk factors associated with HRQoL in patients with inactive and SLE

Disease activity	β	*p *value	Exp (β) (95% confidence interval)
*Inactive*^a^			
Physical health			
Age	− 0.096	0.014	0.909 (0.842–0.981)
Pain			
Disease duration*	− 1.508	0.046	0.221 (0.050–0.976)
Intimate relationships			
Age	− 0.073	0.037	0.930 (0.868–0.996)
Years of education	1.064	0.020	2.899 (1.182–7.109)
Emotional health			
Age	− 0.075	0.032	0.928 (0.867–0.994)
Fatigue			
Age	− 0.109	0.008	0.896 (0.826–0.972)
Current employment status, employed	2.244	0.013	9.429 (1.605–55.395)
*Active*^b^			
Physical health			
SLE flare-ups in the previous six months, yes	− 3.549	0.024	0.029 (0.001–0.622)
Planning			
Age	− 0.081	0.043	0.922 (0.853–0.997)
Intimate relationships			
Age	− 0.182	0.012	0.834 (0.724–0.961)

^a^Inactive SLE was defined by a SLEDAI < 4

^b^Active SLE was defined by a SLEDAI ≥ 4

*The log10-value of the duration of the disease is modeled

## Discussion

This is the first study in Venezuela to measure HRQoL of patients with SLE and to assess the impact of demographic and disease-related characteristics on HRQoL using a valid, reliable, patient-derived, disease-specific questionnaire: the LupusQoL. Our study found that HRQoL of patients with SLE was classified as *better* in all domains of the LupusQoL according to previously established cut-off points for the Venezuelan population with SLE [39]. This is in agreement with results of HRQoL, using the LupusQoL, in patients from Mexico [18] and Peru [34]. It should be taken into account that people with SLE tend to report worse HRQoL compared to general population [13, 15, 40–44], so that even with good general results HRQoL may be affected in specific domains.

Advanced age was correlated with *worse* HRQoL in all domains of the LupusQoL, except in “burden to others”, even though most of the correlations were weak. This was consistent with some studies [17, 18, 34, 45, 46] but not with other ones [33, 47–49]. Naturally, older patients may have *worse* HRQoL due to longer disease duration and higher accrual of disease-related organ damage [50], and increase comorbidities with ageing [51]. More years of education was correlated with *better* “physical health” and “intimate relationships”. We found no description of these correlations in other studies. Longer disease duration was correlated with *worse* “physical health”, “pain”, and “fatigue”. Reports on the correlation between disease duration and “physical health” domain have been varied: some studies reporting no significant correlation [18, 33, 45, 47, 48, 52, 53], while others reporting correlation with better [34, 54] or worse [55] “physical health”. These discrepancies could be due to demographic and disease-related differences among different patient populations. We found no reports on the correlation between disease duration with “pain” and “fatigue” domains in previous studies. Accrued organ damage was correlated with *worse* HRQoL in all domains of the LupusQoL, except in “intimate relationships” and “burden to others”. Except for a study in Brazil [51], others conducted in Mexico [18, 46], Peru [34], the United States [45], the United Kingdom [17], China [41], and Japan [56] showed a similar correlation. Accrued organ damage can affect HRQoL of patients with SLE by pain due to chronic arthritis and the negative effect on physical health, emotional health, and body image due to kidney, lung, central nervous system, and skin diseases, as well as the long-term adverse effects of corticosteroids [45].

Lack of adherence to treatment in patients with SLE ranges between 3 and 76%, depending on the type of medication and the population studied [57]. It is of concern that only 37% of our patients complied with treatment, in consonance with reports from Jamaica [57] and Spain [58]. Patients who fully complied with indicated treatment had higher scores in “physical health” domain compared to patients who did not comply with treatment in at least one of the prescribed medications. Intriguingly, studies from Brazil [59] and China [60] showed that patients with SLE who did not comply with treatment perceived better physical health. In our study, patients with active SLE had higher accrued organ damage score and higher prednisone indication. It is well known that disease activity and accrued organ damage are interrelated variables [42]. Also, the higher the disease activity, the greater the prednisone indication [42] and the greater the daily dosage [61].

We found that disease activity was negatively correlated with all domains of the LupusQoL, except with “intimate relationships” and “burden to others”. Active SLE patients have been reported to engage in physical activity less frequently than recommended by the World Health Organization [62]. Pain is a frequent self-reported symptom in patients with active SLE due to inflammation [63]. Almost all patients with SLE will experience muscle and/or joint pain at a given moment of their disease course, and pain has been reported to contribute to fatigue, anxiety, and depression [63]. Consequently, musculoskeletal symptoms may alter patients’ perceptions in the “physical health”, “body image”, “pain”, and “fatigue” domains [17]. The literature regarding “body image” in patients with active SLE is sparse, and it has been reported to be worse in SLE [17, 64], in accordance with our study. Our study also suggested that “planning” and “emotional health” domains were negatively influenced by the degree of disease activity. It is possible that patients with active SLE experience more fatigue and depression, which impairs their emotional well-being and planning abilities, as has been previously reported [34]. Previous studies have found that advanced age is associated with worse “intimate relationships” [17], which was not consistent with our findings. Our study also found that disease activity did not have an influence on “burden to others”, a domain mainly dependent on the level of social support available to the patient [34], and thus influenced by cultural differences among countries. In summary, our study suggests that patients with active SLE had significantly *worse* “physical health”, “planning”, “emotional health”, “body image”, and “fatigue” compared to patients with inactive SLE.

We found that advanced age, fewer years of education, longer disease duration, having had SLE flare-ups in the previous six months, and being unemployed were risk factors associated with some affected domains of the LupusQoL. This is consistent with results presented in a recent literature review [51]. Advanced age associates with a higher number of comorbidities. Additionally, advanced age patients have experienced longer disease duration, and longer disease duration is also associated with worse HRQoL. Patients with longer disease duration may accrue greater target-organ damage and higher risk of cardiovascular disease [51]. Consistent with our results, previous reports showed worse HRQoL in direct proportion to the number of SLE flare-ups [65, 66]. As previously reported [17], advanced age was a risk factor associated with *worse* “planning” and “intimate relationships” in our patients with active SLE. In turn, in patients with inactive SLE, advanced age was a risk factor associated with *worse* “physical health”, “intimate relationships”, “emotional health”, and “fatigue”. It is possible that, as age increases, the cumulative effect of disease morbidity, comorbidities, drug adverse effects, and worsening of body image may compromise HRQoL to a greater extent than the level of disease activity at a given point in the course of the disease. McElhone et al. [67] documented that all domains of the LupusQoL are sensitive to change with patient-reported deterioration or improvement in health status, with minimum important differences for deterioration ranging from − 2.4 to − 8.7 and for improvement from 3.5 to 7.3. By virtue of the cross-sectional nature of our study, we did not follow-up patients to estimate cut-off points for these minimum important differences, an aspect that will be taken into account for future studies.

Our study has several limitations. First, the number of patients is small. Second, the study is cross-sectional, and the centres involved are tertiary reference centres limiting the generalisability of the results. Third, this study did not screen for fibromyalgia, an entity that can modify patients’ HRQoL. Finally, it is possible that the high proportion of patients who did not comply with indicated treatment in our study, in great measure derived from the critical shortage of drugs amidst the ongoing Venezuelan health crisis [68], may have affected our results. This inference is based on the health system collapse and the reduced access to antimalarials and methotrexate in Venezuela, which has led to the creation of a black market for these drugs, unaffordable for many people [68, 69]. Additional studies are needed with a multicentre and longitudinal design, including other potentially relevant socioeconomic factors to further test the results described in this study.

## Conclusions

Advanced age, fewer years of education, longer disease duration, having had SLE flare-ups in the previous six months, and being unemployed were risk factors associated with *worse* HRQoL in our patients. Disease activity was negatively correlated with most domains of the LupusQoL. Our study provides information that could be used to report health policies and improve the quality of medical care provided to patients with SLE through interventions to favourably influence each of the HRQoL domains.

## Data Availability

All data generated or analysed during this study are included within this article.

## Abbreviations

SLE: Systemic lupus erythematosus
HRQoL: Health-related quality of life;
LupusQoL: Lupus Quality of Life
SLEDAI: Systemic Lupus Erythematosus Disease Activity Index
SLICC-DI: Systemic Lupus International Collaborating Clinics Damage Index
